# Could the Early Detection of Atrial Fibrillation Reduce the Risk of Developing Dementia?

**DOI:** 10.3390/biomedicines12081931

**Published:** 2024-08-22

**Authors:** Fabrice Demoniere, Rim Abdelli, Léna Rivard

**Affiliations:** Montreal Heart Institute, Université de Montréal, 5000 Belanger Street, Montreal, QC H1T 1C8, Canada

**Keywords:** atrial fibrillation, dementia, cognitive decline, anticoagulation, AF ablation, prevention, stroke, integrative approaches

## Abstract

Atrial fibrillation (AF) and dementia are major global public health issues and share common risk factors, especially after the age of 65 and regardless of the presence of stroke. Despite accounting for potential confounders, AF appears to be an independent risk factor for cognitive decline and dementia. The mechanisms are likely to be multifactorial and may include AF-related ischemic stroke, cerebral hypoperfusion, microbleeds, systemic inflammation, genetic factors, and small vessel disease, leading to brain atrophy and white matter damage. The early aggressive management of AF and comorbidities may reduce the risk of dementia. Indeed, the early detection of AF-related cognitive impairment should allow for the early implementation of measures to prevent the development of dementia, mainly through integrative approaches involving the correction of risk factors and maintenance of rhythm control. Well-designed prospective studies are needed to determine whether early detection and AF treatment can prevent dementia and identify whether optimal integrative measures are effective in preventing cognitive impairment and dementia.

## 1. Impact of Atrial Fibrillation and Dementia

**Figure 1:**
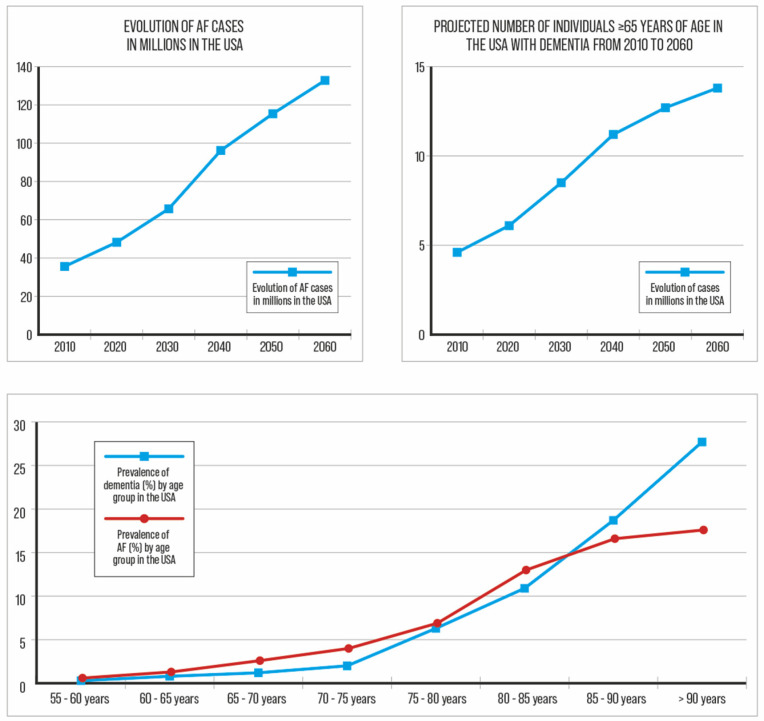
Projections of dementia and AF in the USA.

The increasing prevalence and incidence of atrial fibrillation (AF), stroke, and dementia are major public health issues (Figure 1). In 2010, 35.6 million people in the United States of America (USA) had dementia, and this number is expected to reach 65.7 million by 2030 and 115.4 million by 2050 [1]. The worldwide prevalence of dementia is expected to increase by 100% to 250% between 2019 and 2050 [1,2]. Similarly, AF, the most common cardiac arrhythmia, affecting more than 37 million people globally, is expected to increase [3]. (Figure 1). Atrial fibrillation is associated with increased mortality and morbidity, including a 5-fold increased risk of stroke, and shares the same epidemiological evolution and risk factors as dementia [3,4]. Clinical AF is associated with a higher incidence of all types of dementia [5,6,7]. Thus, the early detection of AF-related cognitive impairment should theoretically allow for integrative approaches to prevent the development of dementia.

## 2. Cognitive Function and Dementia

Cognitive functions include domains such as attention, memory, and executive functions that combine working memory, flexible thinking, self-control, language, and visuospatial processing. Atrial fibrillation patients showed worse cognitive performance for the attention and executive function domains but no significant difference in memory functions [8,9,10]. Cognitive decline is defined as changes in cognitive function that are greater than expected from normal aging, diagnosed by changes in standardized cognitive tests, and is classified into five categories. The first category is a cognitive decline that does not affect daily activities. Dementia, the fifth category, is defined as deficits in at least “2 cognitive domains that are more important than previous levels of functioning and are severe enough to affect activities of daily living” [11]. The diagnosis of dementia according to the classification schemes (DSM-II, DSM-III-R, DSM-IV, ICD-9, or CAMDEX) can be challenging, and the prevalence of dementia in different cohorts varied from 3.1 to 29.1% depending on the definition used [12]. The Montreal Cognitive Assessment (MoCA) and the Mini-Mental State Examination (MMSE) are the most commonly used screening tools to detect mild cognitive impairment, but the diagnosis of dementia requires confirmation in a memory or neurology clinic with a highly specialized activity [13,14]. The most common forms of dementia are Alzheimer’s disease (AD) and vascular dementia. Other types include mixed dementia, dementia with Lewy bodies, and frontotemporal dementia. 

## 3. Shared Confounders

**Figure 2:**
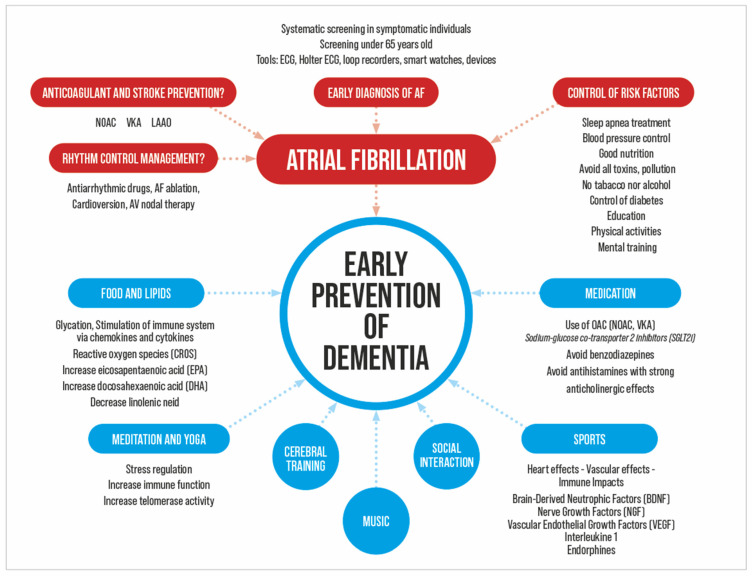
Potential targets to decrease dementia in AF patients.

Atrial fibrillation and dementia share common risk factors (Table 1 and Figure 2) [7]. Age is the most important risk factor for both dementia and AF and is a major confounding factor. Experimental studies have shown that high blood pressure can cause changes in inflammation, fibrosis, and hypertrophy of the left atrium, thereby inducing AF [15]. Hypertension damages the intracerebral microcirculation and impairs cerebral function. In hypertensive patients, cognitive impairment was statistically more prevalent in those with AF than in those without AF [16]. The Swedish cohort study of 421,855 patients with type 2 diabetes reported a 35% increased risk of AF in patients with type 2 diabetes, with an additional increased risk in patients with poorer glycemic control, glycemic variability, and a longer duration of diabetes [17]. It has been suggested that six sleep conditions (insomnia, fragmentation, daytime dysfunction, prolonged latency, rapid eye movement sleep behavioural disorder, and excessive time in bed) may be moderate-to-high risk factors [18]. for dementia. Obstructive sleep apnea disrupts autonomic nervous system balance and increases the risk of developing AF [19]. Sleep apnea induces brain hypoxia with deficits in executive function, attention, and memory [20]. Obesity increases the risk of developing AF metabolic syndrome [21]. Excess body fat tissue has been found to be pro-inflammatory and may be linked to dementia [22]. Physical inactivity is significantly associated with an increased risk of developing dementia [23] but the effect on AF remains controversial [24]. Smoking is associated with the neuropathology of dementia and AD by increasing the concentration of free radicals associated with oxidative damage to membrane lipids and neuronal, glial, and vascular tissues of the brain [25]. Heavy alcohol consumption is a risk factor both for AF through atrial remodelling [26] and for cognitive impairment through direct toxicity and indirectly through trauma. Chronic kidney disease increases the risk of developing heart disease and AF [27] and is associated with higher levels of cerebral uremic toxins, which contribute to neurodegeneration [28]. Hyperthyroidism is a well-established risk factor for AF [29]. Anxiety, depression, or preoccupation with symptoms is known to increase the risk of dementia and affects 20–50% of people with AF [30,31].

## 4. Suspected Mechanisms

The relationship between AF and cognitive decline is complex and likely to be multifactorial. Small vessel disease, cerebral infarcts, decreased cerebral perfusion, cerebral injury, and the disruption of the blood–brain barrier are the most likely putative mechanisms [7]. Atrial fibrillation is associated with a 4- to 5-fold increased risk of ischemic stroke and a 2.6-fold increased risk of silent cerebral infarct [5]. Reported rates of new-onset dementia are as high as 24% within 3 years after stroke and 33% within 5 years [32]. In the Atherosclerosis Risk in Communities (ARIC) study, after a 20-year follow-up, 2,106 participants developed AF and 1,157 developed dementia. After adjustments for cardiovascular risk factors, including clinically overt ischemic stroke, the average decline in cognitive function was greater in participants with AF compared with those without AF. Interestingly, only stroke-free AF patients with silent cerebral infarcts suffered from cognitive decline [33]. White matter lesions (abnormal myelination areas) represent 18% of brain imaging findings in patients with AF after 2 years of follow-up [34]. In the Swiss AF Study, 1,227 AF patients (mean CHAD_2_DS_2_VASc score 3.0) were enrolled in a prospective, multicentre cohort study [35]. During a 2-year follow-up, 2.3% had a stroke or transient ischemic attack (TIA) and 5.5% had a silent infarct. Other studies reported large infarcts in 22% of participants, small non-cortical infarcts in 21%, microbleeds in 22%, white matter lesions in 18.7%, and smaller hippocampal volumes [8]. Cerebral microbleeds are also more often seen in patients with AF compared to those in sinus rhythm. 

Hypoperfusion associated with AF may also induce ischemic demyelination similar to that seen in cerebral small vessel disease, promoting cognitive decline. Several studies have suggested that inflammation is an important component of the pathophysiological process leading to AF and that AF, in turn, exacerbates the inflammatory response [36,37]. Inflammation is thought to increase hypercoagulability and thrombus formation, potentially increasing the risk of stroke and the cerebrovascular dysregulation associated with AD and vascular dementia [38,39].

Atrial fibrillation is associated with elevated levels of C-reactive protein (CRP), interleukin (IL-2, IL-6, and IL-8) and other inflammatory markers (tumour necrosis factor alpha; TNF-α) that may increase oxidative stress and endothelial injury [40]. Galenko et al. reported an increase in circulating biomarkers of neuronal and glial injury Tau and GFAP in patients with AF compared to patients in sinus rhythm [41]. Endothelial dysfunction in AF is characterized by increased coagulation activity with higher levels of D-dimer, fibrinogen, prothrombin fragments 1 and 2, platelet factor-4, thromboglobulin, and Von Willebrand factor [42]. In animal research, the relationship between inflammation and dementia appears to be mediated by sphingosine-1-phosphate (S1P) [43]. Sphingosine-1-phosphate induces vasoconstrictive effects on cerebral arteries with a myogenic response in smooth muscle cells and leads to critical hypoperfusion of the brain [44].

The ε4 allele of the apolipoprotein APOE is the strongest risk factor associated with AD [45,46]. ε4 APOE homozygotes have a 12-fold increased risk [25] of AD. The ApoE4 phenotype is significantly higher in AF patients, but causality between this gene and AF has not been confirmed [45]. Three rare single-gene variants are known to cause early AD: amyloid precursor protein (APP) on chromosome 21, presenilin 1 (PSEN1) on chromosome 14, and presenilin 2 (PSEN2) on chromosome 1. However, these genes do not appear to be more common in AF patients [47]. In AF patients, those who express the paired-like homeodomain transcription factor 2 (PITX2) gene appear to have an increased risk of cerebral ischemic events and dementia. The PITX2 gene has been associated with AF as alterations in this gene cause abnormal atrial electrical activity [47]. In a cohort of 112 Caucasian patients, the PITX2 gene was significantly associated with AF patients with dementia compared to AF patients without dementia (p = 0.008) [48]. During normal sinus rhythm, end organs are perfused with stable pulsatility, whereas during AF, the irregular rhythm results in variable diastolic filling times and decreased cardiac output (17% during exercise in AF patients) [49]. An irregular AF rhythm favours chronic brain hypoperfusion as described in heart failure [50,51]. Near-infrared spectroscopy systems measure changes in oxyhemoglobin to estimate blood flow, and a significant decrease in frontal brain perfusion, associated with a 37% decrease in frontal brain activity, has been described in AF patients while performing specific cognitive tasks [51,52]. 

## 5. Causality or Shared Risk Factors

The association between AF and cognitive impairment/dementia appears to persist even after adjustments for known risk factors [7,53] and is stronger in younger patients compared with older patients with a higher burden of common risk factors, which would not be expected if the association were due to confounding alone [7]. Other factors that favour causality are temporality (AF precedes cognitive decline) and a biological gradient between AF burden and cognitive impairment [54,55,56]. Furthermore, studies have shown that left atrial dysfunction and left atrial size correlate significantly with risk cognitive impairment [57]. This suggests a link between AF, atrial cardiomyopathy, and cognition. Thus, there is a potential value of early AF detection, but it is not yet known whether AF detection and integrated approaches would prevent cognitive decline. This will depend in part on the results of ongoing trials, particularly with regard to the effect of OACs on cognitive outcomes.

## 6. Rhythm Control Strategies

Several longitudinal and case–control studies have reported a benefit of AF ablation on cognition [58,59]. The incidence of all types of dementia, except for vascular dementia, decreased in patients with AF who underwent catheter ablation. The prospective Intermountain Atrial Fibrillation study included 16,848 patients. Over a 3-year period, new-onset AD occurred in 0.2% of the AF patients who underwent left atrial catheter ablation compared with 0.9% of the patients who did not undergo ablation and 0.5% of the non-AF patients (p < 0.001) [58]. Other forms of incident dementia occurred in 0.4%, 1.9%, and 0.7% of the ablated AF patients, non-ablated AF patients, and non-AF patients, respectively (p < 0.001). Yoshihisa reported increased blood flow in the frontal and temporal brain regions 3 months after AF ablation [60]. However, in the prospective impact of atrial fibrillation burden on cognitive function after left atrial ablation (MACPAF) study, AF burden had no significant impact on overall cognitive performance within 6 months after ablation in 30 symptomatic paroxysmal AF patients [61]. In a recent meta-analysis, catheter ablation was associated with a lower risk of a subsequent diagnosis of dementia compared to medical therapy [62]. The ongoing DIAL-F trial (Cognitive Impairment in Atrial Fibrillation, NCT01816308) is testing the hypothesis that catheter ablation can prevent cognitive decline. The results of the primary endpoint indicate an improvement or no worsening of the MoCA score in two groups (antiarrhythmic drugs (AADs) vs. AF ablation) during a 2-year follow-up. However, left atrial ablation is also associated with an increased risk of stroke and silent brain infarcts, which may result in long-term neurocognitive impairment [63,64]. The risk of TIA and stroke after AF ablation has been estimated at 0.5−1% and the prevalence of silent cerebral infarction at 7–14% in magnetic resonance imaging studies. The study by Medi et al. shows a subtle post-procedural cognitive dysfunction after AF ablation in 150 patients, which correlates with the time spent in the left atrium [64]. The ongoing AFCOG trial (Acute cognitive changes during atrial fibrillation episodes, NCT04033510) includes a cohort of 600 patients with a documented history of AF and is evaluating their cognitive performances in AF and sinus rhythm during follow-up. A sub-study of the AFFIRM trial (Atrial Fibrillation Follow-up Investigation of Rhythm Management) showed no difference in MMSE scores in 245 patients during a mean follow-up of 3.5 years [65]. After a 10-week follow-up post-electrical cardioversion, cerebral blood flow increased by 5% in patients who remained in sinus rhythm compared to patients still in AF in a small study of 44 patients [66]. 

## 7. Anticoagulation

Several observational studies have demonstrated a reduction in cognitive impairment and dementia in patients receiving anticoagulants (OACs) compared with those not receiving OAC therapy. [67,68,69,70,71] Therefore, OACs may be an important factor in the reduction in dementia incidence. It remains unknown whether the effect of vitamin K antagonists (VKAs) or non-vitamin K antagonist oral anticoagulants (NOACs) on cognition is different. Non-vitamin K antagonist oral anticoagulants were associated with a 9% lower risk of incident dementia compared with VKA in an observational study [70]. In the CAF trial (Impact of anticoagulation therapy on the cognitive decline and dementia in patients with nonvalvular atrial fibrillation trial), the use of dabigatran and well-managed warfarin therapy were associated with similar risks of stroke, cognitive decline, and dementia at 2 years in 120 participants aged >65 years [72]. Several studies are underway that will help understand the role of OAC and left atrial appendage occlusion (LAAO) in reducing the risk of dementia (Figure 2). The ARISTA trial (Trial of Apixaban Versus Warfarin in Reducing Rate of Cognitive Decline, Silent Cerebral Ischemia and Cerebral Microbleeds in Patients With Atrial Fibrillation, NCT03839355) is testing the hypothesis that apixaban reduces the rate of decline in cognitive function compared to VKA. The benefit of OAC in patients with AF without a clear indication for OAC therapy remains unknown. The BRAIN-AF trial (Blinded Randomized Trial of Anticoagulation to Prevent Ischemic Stroke and Neurocognitive Impairment in Atrial Fibrillation, NCT02387229) is currently testing the efficacy of OAC therapy in patients with AF at a low risk of stroke [73]. This prospective, multicentre, double-blind, randomized controlled trial is evaluating rivaroxaban (15 mg daily) on the composite outcome of stroke/TIA or neurocognitive decline (defined as a decline in the MoCA score ≥ 3). The ongoing OCEAN trial (Optimal Anticoagulation for Higher Risk Patients Post-Catheter Ablation for Atrial Fibrillation Trial, NCT02168829) is comparing rivaroxaban (15 vs. ASA 81 mg) in patients without clinical AF recurrences 12 months after AF ablation for the combined endpoint stroke, systemic embolism, and cognitive decline (MoCA and MMSE). Mohanty et al. compared cognitive status in 98 patients who underwent LAAO or remained on OAC after AF ablation. At 1 year, continued OAC therapy was associated with worsening cognitive function compared to LAAO regardless of sex and AF type (−3.38 in MOCA; 95% CI, −4.75 to −2.02; p < 0.0001) [74]. The PLUG trial (Overall and MRI-based Impact of Percutaneous Left Atrial Appendage Closure on the Cognitive Decline and Dementia in Patients With AF, NCT03091855) is evaluating the role of LAA closure in reducing the risk of dementia. 

## 8. Integrated Approaches

The integrated approach consists of four points, including patient assessment, risk factor management, anticoagulation, and rhythm management. Modifiable factors include cardiovascular risk factors, psychiatric factors, diet, lifestyle, and education [75]. Integrative measures in the prevention and care of dementia aim to maximize cognition and improve quality of life through effective interventions such as physical exercise, intellectual stimulation, or leisure activities to reduce the risk of dementia in later life [76]. The FINGER (Finnish Geriatric Intervention Study to Prevent Cognitive Impairment and Disability) trial randomized 1,260 individuals older than 60 years of age at high risk for dementia to intervention (n = 631) or control (n = 629) [77]. The intervention group consisted of nutritional counselling, exercise, cognitive training, social activities, and the management of metabolic and vascular risk factors. These individuals showed a mean improvement in a composite cognitive measure of executive function and processing speed after 2 years of follow-up. Less cognitive reserve appears to lead to the earlier development of dementia [78,79]. Building brain reserve early in life through education levels and intellectual stimulation could promote cognitive resilience in later life and decrease the rate of dementia [75,78,80]. Modifiable factors are mainly distributed between midlife (age 45–65 years) and later life (age > 65 years). In midlife, the weighted population attributable fractions (PAFs) are 2% for hypertension, 0.8% for obesity, and 9.1% for hearing loss. In later life, the PAF is 5.5% for smoking, 4.0% for depression, 2.6% for physical inactivity, 2.3% for social isolation, and 3.2% for diabetes [76]. To prevent dementia, the National Institutes on Aging at the National Institutes of Health (NIH) recommends controlling high blood pressure, following a healthy diet (mix of fruits and vegetables, whole grains, lean meats and seafood, unsaturated fats such as olive oil, low-fat or fat-free dairy products, and less consumption other fats and sugars), maintaining a healthy weight, and staying physically active (at least 150 min of moderate-intensity physical activity each week) [81]. The study by Bherer et al. found a significant improvement in cognition in patients after 20 weeks of aerobic and resistance training [82]. Quality and quantity of sleep are also modifiable factors; 35% of the population sleeps less than 7 h a night, whereas 8 h of sleep a night is recommended. Sleep disorders like sleep apnea should also be corrected. Other factors include preventing head injuries, taking steps to prevent falls, avoiding or limiting alcohol, stopping tobacco use, and avoiding some medications (Figure 2). In the retrospective study by Proietti et al., SGLT2i reduced the risk of cerebrovascular events, incident dementia, heart failure, and death in patients with concomitant AF and diabetes mellitus type 2 [83].

## 9. Potential Benefits of Early AF Detection 

**Figure 3:**
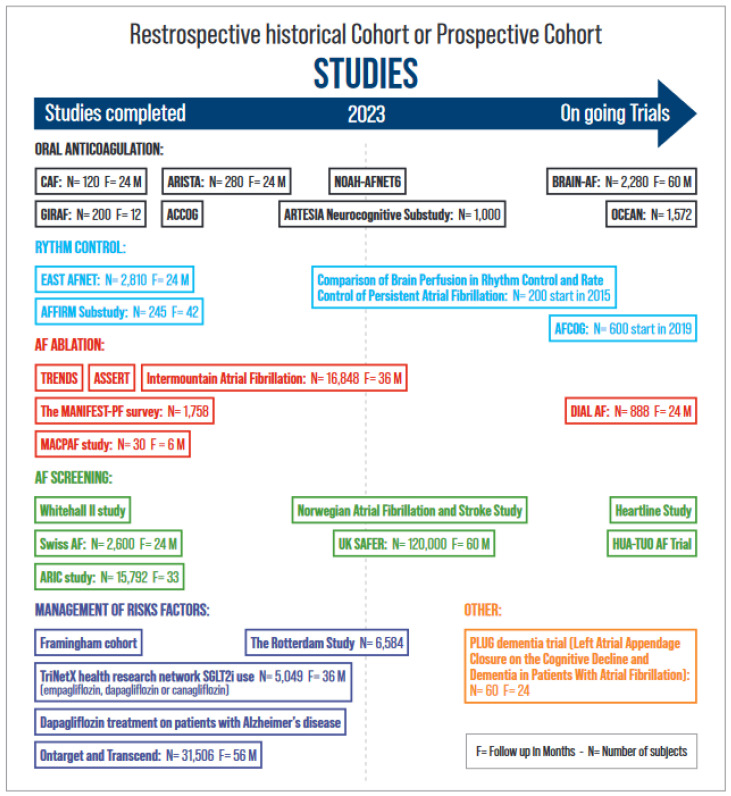
Published and ongoing trials on AF and cognition.

Since AF increases the risk of dementia, its early diagnosis and treatment should theoretically reduce the risk of cognitive impairment. The target population that should be screened has yet to be established and will depend on the results of ongoing trials. Similarly, ideal strategies for screening for AF remain to be defined. The technology is constantly evolving with the development of technologies such as ECG snapshots, continuous external wearables (smartwatches, rings, or phones), or implanted devices (loop recorders or other devices). There is increasing evidence that the early aggressive management of AF and comorbidities reduces the risk of dementia. Results from the Korean nationwide registry examining the associations of blood pressure with dementia risk in midlife AF patients with incident AF suggest that aggressive blood pressure control and OAC are associated with a decreased incidence of dementia after 6 years of follow-up [84]. From the same large database, Yang et al. compared AF patients who met all ABC pathway criteria (adequate stroke prevention, medical follow-up, and management of comorbidities) with those who did not. An ABC-integrated care approach to AF management was associated with a lower risk of dementia [85]. The EAST-AFNET 4 (Early Treatment of Atrial Fibrillation for Stroke Prevention) trial demonstrated a 21% lower risk of cardiovascular outcomes in 1395 patients randomized to early rhythm control therapy compared to usual care [86]. The Whitehall II study demonstrates that the early age of AF and longer exposure to AF are associated with a faster cognitive decline or dementia [87]. The prospective Intermountain Atrial Fibrillation study reports that OAC combined with AF ablation performed in patients younger than 65 years reduces the risk of new-onset AD [53]. The use of OAC therapy started within 1 year of initial AF diagnosis appears to reduce the risk of dementia [88,89]. Ongoing trials will fill in the gaps and answer questions about the role of integrated approaches, OAC, rhythm control, rate control, and AF screening (Figure 3). At present, it is not known whether AF screening should be extended to all asymptomatic individuals with risk factors for stroke to detect subclinical AF and prevent not only stroke but also dementia [73,90]. Biomarkers and genetics can help define individuals at risk for dementia and AF. Advances in Alzheimer’s biomarker identification and neuroimaging make it possible to detect beta-amyloid accumulation in the brain [91]. B-type natriuretic peptide (NT-proBNP) is a predictor of AF in healthy patients without heart failure when the BNP rate exceeds 615 pg/mL [92]. 

## 10. Conclusions and Future Directions

Several studies have shown that AF is associated with an increased risk of dementia, independent of clinical stroke, with the strongest association in younger participants and with longer AF duration. However, the relationship between AF and dementia remains difficult to establish because of the many confounding factors common to both conditions. Several pathophysiological mechanisms have been proposed, some of which may be amenable to early intervention (OAC, the role of LAAO, and rate and rhythm control). Prospective and randomized clinical trials are needed to understand the relationship between AF and cognitive decline and to determine which interventions can effectively prevent cognitive decline and avert or delay the onset of dementia. 

## Figures and Tables

**Table 1:** Main shared risk factors.

Main Shared Risk Factors
Age
Arterial hypertension
Diabetes mellitus
Coronary artery disease
Excessive alcohol consumption
Heart failure
Hyperlipidemia
Obesity and Western diet
Physical inactivity
Sleep apnea and chronic sleep deprivation

## Abbreviations

AF	Atrial fibrillation
AADs	Antiarrhythmic drugs
AD	Alzheimer’s disease
AFCOG	Acute cognitive changes during atrial fibrillation episodes
AFFIRM	Atrial Fibrillation Follow-up Investigation of Rhythm Management
ARIC	Atherosclerosis Risk in Communities
ARISTA	Trial of Apixaban vs warfarin in reducing Rate of cognitive decline, silent cerebral ischemia and cerebral microbleeds in patients with atrial fibrillation
BNP	B-type natriuretic peptide
BRAIN-AF trial	Blinded Randomized Trial of Anticoagulation to Prevent Ischemic Stroke and Neurocognitive Impairment in Atrial Fibrillation
CAF trial	Impact of anticoagulation therapy on the cognitive decline and dementia in patients with nonvalvular atrial fibrillation
CHA2DS2VASc score	Congestive heart failure (1 point), Hypertension (1 point), Age ≥ 75 (2 points), Diabetes (1 point), prior Stroke or TIA (2 points), Vascular disease (1 point), Age 65–74 (1 point), and Sex (female; 1 point)
EAST-AFNET 4	Early Treatment of Atrial Fibrillation for Stroke Prevention Trial 4
FINGER	Finnish Geriatric Intervention Study to Prevent Cognitive Impairment and Disability
LAAO	Left atrial appendage occlusion
MACPAF	Prospective Impact of atrial fibrillation burden on cognitive function after left atrial ablation study
MMSE	Mini-Mental State Examination
MoCA	Montreal Cognitive Assessment
NOACs	Non-vitamin K antagonist oral anticoagulants
OAC	Oral anticoagulation
OCEAN trial	Optimal Anticoagulation for Higher Risk Patients Post-Catheter Ablation for Atrial Fibrillation Trial
PLUG	Overall and MRI-based Impact of Percutaneous Left Atrial Appendage Closure on the Cognitive Decline and Dementia in Patients With AF
PAFs	Population attributable fractions
TIA	Transient ischemic attack
USA	United States of America
VKA	Vitamin K antagonist
